# Theoretical Investigation of Ca^2+^ Intercalation in WS_2_ as a Negative Electrode Material for Calcium-Ion Batteries: Supported by Experimental Evaluation

**DOI:** 10.3390/ijms26168005

**Published:** 2025-08-19

**Authors:** Seunga Yang, SangYup Lee, Paul Maldonado Nogales, Yangsoo Kim, Soon-Ki Jeong

**Affiliations:** 1 Department of Future Convergence Technology, Graduate School, Soonchunhyang University, Soonchunhyang-ro 22-gil, Sinchang-myeon, Asan-si 31538, Chungcheongnam-do, Republic of Korea; tmddk1107@sch.ac.kr (S.Y.); 20237450@sch.ac.kr (S.L.); maldonado@sch.ac.kr (P.M.N.); 2 Korea Basic Science Institute, Jeonju Center, Jeonju-si 54907, Jeollabuk-do, Republic of Korea; 3 Department of Energy Engineering, Soonchunhyang University, Soonchunhyang-ro 22-gil, Sinchang-myeon, Asan-si 31538, Chungcheongnam-do, Republic of Korea; 4 Advanced Energy Research Center, Soonchunhyang University, Soonchunhyang-ro 22-gil, Sinchang-myeon, Asan-si 31538, Chungcheongnam-do, Republic of Korea

**Keywords:** Ca-ion batteries, structural stability, first-principles calculations, orbital interactions, WS_2_ (tungsten disulfide)

## Abstract

Tungsten disulfide (WS_2_), a two-dimensional layered material with favorable electronic properties, has been explored as a promising negative electrode material for calcium-ion batteries (CIBs). Despite its use in monovalent systems, its performance in divalent Ca^2+^ intercalation remains poorly understood. Herein, a combined theoretical and experimental framework is used to elucidate the electronic mechanisms underlying Ca^2+^ intercalation. Theoretical insights were obtained through density functional theory calculations, incorporating periodic simulations using the Vienna Ab initio Simulation Package, and localized orbital-level analysis using the discrete variational Xα method. These approaches reveal that Ca^2+^ insertion induces significant interlayer expansion, lowers diffusion barriers, and narrows the bandgap compared to Li^+^. Orbital analysis revealed strengthened W–S bonding and diminished antibonding interactions, which may contribute to the improved structural resilience. Electrochemical tests validated these predictions; the CaWS_2_ electrode delivered an initial discharge capacity of 208 mAh·g^−1^ at 0.1C, with 61% retention after 50 cycles at 1C. The voltage profile exhibits a distinct plateau near 0.7 V, consistent with a two-phase-like intercalation mechanism, contrasting with the gradual slope observed for Li^+^. These findings suggest that Ca^2+^ intercalation facilitates both rapid ion transport and enhanced structural robustness. This study offers mechanistic insights into multivalent-ion storage and supports the design of high-performance CIB electrodes.

## 1. Introduction

The increasing demand for electric vehicles and grid-scale energy storage systems has intensified efforts to develop next-generation rechargeable batteries with high energy densities, extended cycle lives, and improved safety. Although lithium-ion batteries remain the dominant commercial technology, concerns over their limited lithium availability, rising material costs, and safety risks challenge their long-term sustainability [1,2,3]. These issues have motivated the exploration of alternative battery chemistries that are both resource-abundant and capable of delivering competitive electrochemical performance [4,5,6].

Among the emerging alternatives, calcium-ion batteries (CIBs) have gained increasing attention because of their natural abundance, low cost, and divalent charge, enabling two-electron transfers per ion and a high theoretical capacity [7,8,9]. In addition, calcium has a standard reduction potential of −2.87 V (vs. SHE), thereby supporting the higher operating voltages. Together, these factors offer the potential for high-energy-density CIB systems. However, the practical implementation of CIBs remains limited by the sluggish Ca^2+^ diffusion kinetics arising from the ion’s large size and strong electrostatic interactions with host materials [10,11,12,13]. In addition, Ca^2+^ insertion can induce structural disruption and interfacial instability, making it challenging to achieve reversible and durable calcium storage. These limitations underscore the necessity for host materials capable of withstanding divalent cations while maintaining structural integrity throughout repeated cycling [14,15,16]. To address these challenges, recent studies have focused on low-dimensional materials with favorable structural and electronic characteristics that can support multivalent-ion insertion [17,18,19,20].

Two-dimensional layered materials, in particular, offer promising platforms for multivalent-ion storage due to their interlayer channels and adjustable bonding characteristics [21,22,23,24,25]. In particular, tungsten disulfide (WS_2_) is a representative transition-metal dichalcogenide with a large interlayer spacing, chemical stability, and favorable electronic conductivity [26,27,28,29,30]. Although its behavior under lithium and sodium intercalation has been well studied [31,32,33,34], the calcium-ion-intercalation mechanism in WS_2_ remains largely unexplored. To the best of our knowledge, no previous studies have investigated WS_2_ as a CIB negative electrode material using an integrated theoretical and experimental approach. This study aimed to fill this gap.

In this study, the potential of WS_2_ as a calcium-ion host was evaluated using density functional theory (DFT) calculations, including periodic simulations based on the Vienna ab initio simulation package (VASP) and orbital-level analysis using the discrete variational Xα (DV-Xα) method. While the VASP-based approach provides insight into the structural evolution within a periodic supercell framework, the DV-Xα method complements this by resolving the localized bonding characteristics and charge redistribution at the orbital level. The structural deformation, charge-transfer behavior, and electronic modifications induced by Li^+^ and Ca^2+^ intercalation were analyzed. Galvanostatic cycling and cyclic voltammetry measurements were performed to validate theoretical predictions. This dual theoretical–experimental framework revealed that Ca^2+^ intercalation leads to greater interlayer expansion, reduced diffusion barriers, and improved electronic conductivity. The enhanced transport behavior and bonding stabilization suggest that WS_2_ could serve as a structurally resilient platform for advanced multivalent-ion batteries.

## 2. Results and Discussion

### 2.1. Structural Effects of Li^+^ and Ca^2+^ Intercalation in WS_2_ Clusters

**Figure 1:**
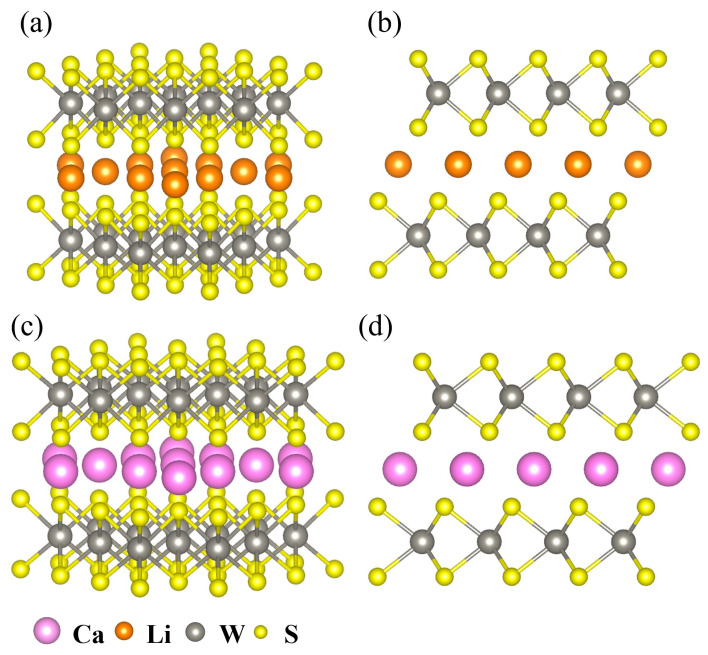
Atomic structures of ion-intercalated WS_2_ models. (**a**,**b**) Top and side views of Li^+^-intercalated WS_2_; (**c**,**d**) corresponding views of Ca^2+^-intercalated WS_2_. Compared to Li^+^, Ca^2+^ intercalation causes a larger interlayer expansion, reflecting its divalent nature and larger ionic radius.

To clarify the structural effects specifically induced by Ca^2+^ insertion, Li^+^ intercalation was also examined as a reference, given its partially reported behavior in WS_2_. This comparison allows for a more systematic distinction between the monovalent and divalent ion-intercalation mechanisms within the same host framework. Finite cluster models were constructed from the VASP-optimized WS_2_ structures to preserve the 2H-phase layered symmetry. These models were then used in the DV-Xα calculations to gain atomistic insight into ion–host interactions, as illustrated in Figure 1. Li^+^ and Ca^2+^ ions were inserted into the interlayer space along the c-axis, with the W and S atoms arranged symmetrically to retain the trigonal prismatic coordination. In this study, both ions were modeled in their desolvated state, as commonly assumed for layered transition-metal dichalcogenides such as MoS_2_ and TiS_2_, where the interlayer structure allows them to completely shed their solvation shells prior to intercalation. This model enables a direct comparison of ion–host interactions without the confounding influence of solvent shielding effects. The structural differences between Li^+^ and Ca^2+^ intercalation are highlighted in Figure 1a–d. Figure 1a,b show the top and side views of the Li-intercalated WS_2_ cluster, respectively, whereas Figure 1c,d show the corresponding views of the Ca-intercalated system. Notably, Ca^2+^ insertion caused a greater expansion of the interlayer spacing than that of Li^+^. This structural change is expected to enhance calcium-ion mobility, which is an essential factor for achieving high-rate electrochemical performance. This difference is visually evident; Li^+^ ions align in a single row between the WS_2_ layers, whereas Ca^2+^ ions occupy a broader interlayer region. This reflects their large coordination radii and weak polarizing interactions. This expansion involves not only geometric changes but also notable modifications in the electronic structure, reflecting a stronger lattice response and charge redistribution induced by divalent-ion insertion.

As summarized in Table 1, the pristine WS_2_ structure exhibits lattice parameters of a = 3.15 Å and c = 12.32 Å, which are in good agreement with previously reported experimental values [35,36]. Upon Li^+^ intercalation, the a-axis expands slightly to 3.17 Å, and the interlayer spacing increases to 13.04 Å, indicating moderate lattice relaxation. In contrast, Ca^2+^ intercalation leads to a more significant increase in both directions, with lattice parameters of a = 3.53 Å and c = 13.09 Å. This pronounced expansion is attributed to the larger ionic radius of Ca^2+^ (1.02 Å) compared to that of Li^+^ (0.76 Å). The size difference increased the interlayer repulsion, leading to a more pronounced lattice expansion, particularly along the a-axis. While this difference is largely size-driven, the greater extent of structural relaxation suggests the redistribution of bonding interactions and electrostatic forces within the host lattice. Nevertheless, all the optimized structures preserve the 2H-phase crystallographic symmetry (space group P6_3_/mmc, No. 194), allowing reliable comparisons across different intercalation states.

To evaluate local bonding environments, multiple cluster compositions were tested: XW_2_S_12_, X_7_W_12_S_24_, X_7_W_12_S_48_, X_13_W_12_S_48_, and X_13_W_24_S_48_ (X = Li or Ca). Among these, the X_13_W_24_S_48_ cluster was selected for further analysis because it maintained a W:S ratio of 1:2 after ion insertion. This composition preserves formal charge neutrality and improves the fidelity of electronic structure modeling. Madelung potentials were applied to assign oxidation state-based charges to each atom: +1 for Li, +2 for Ca, +4 for W, and −2 for S. This charge distribution reflects the ionic nature of the layered WS_2_ and ensures consistency in the bonding and electronic analyses. Although interlayer expansion promotes ionic diffusion, it may also introduce mechanical softness, potentially compromising the structural integrity during extended cycling. Thus, balancing ionic conductivity with mechanical robustness remains critical in the design of multivalent-ion battery electrodes.

### 2.2. Electronic Structure and Charge Transfer Characteristics of WS_2_ Clusters

**Figure 2:**
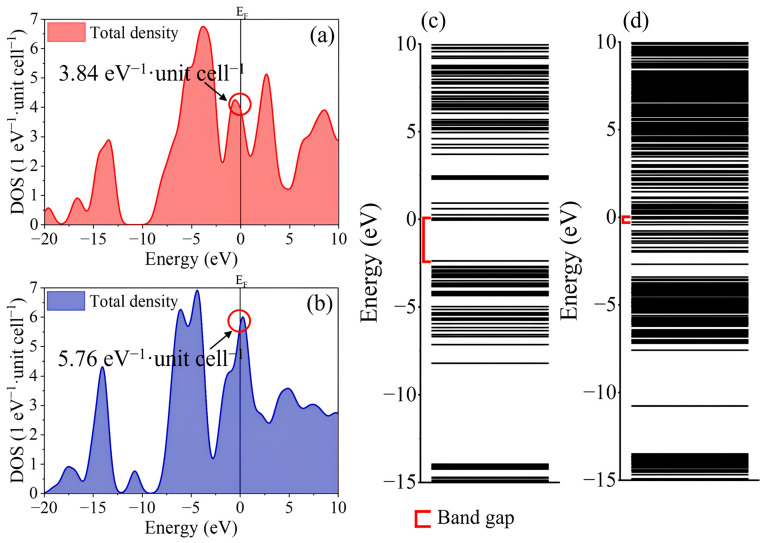
Total DOS and MO energy levels of ion-intercalated WS_2_ clusters. (**a**,**b**) Total DOS of LiWS_2_ and CaWS_2_, with CaWS_2_ exhibiting a higher DOS at the Fermi level (5.76 vs. 3.84 eV^−1^·unit cell^−1^); (**c**,**d**) MO energy level diagrams showing reduced bandgap in CaWS_2_, indicating enhanced electronic conductivity upon Ca^2+^ intercalation. The red brackets in (**c**,**d**) indicate the energy gap between the valence band maximum and the conduction band minimum.

To further explore the electronic and electrochemical properties of WS_2_ under ion intercalation, total and partial density of states (DOS), molecular orbital (MO) energy levels, electron transfer numbers, and the open-circuit voltages (OCVs) were analyzed [37,38]. As shown in Figure 2a,b, the total DOS of CaWS_2_ showed a substantially higher electronic state density at the Fermi level (E_F_) than that of LiWS_2_, indicating an improved intrinsic electronic conductivity. Specifically, CaWS_2_ exhibits a total DOS of 5.76 eV^−1^·unit cell^−1^ near E_F_, compared to 3.84 eV^−1^·unit cell^−1^ for LiWS_2_ This increased DOS suggests a higher density of low-energy electronic states, which may lower transport barriers and facilitate electronic conduction. The elevated DOS near E_F_ in CaWS_2_ also indicates enhanced charge delocalization, which likely promotes higher carrier mobility and more efficient redox activity during cycling.

The MO energy-level diagrams (Figure 2c,d) further illustrate that CaWS_2_ exhibits a narrower bandgap and more densely distributed energy levels near E_F_, suggesting lower activation barriers for electronic excitation and a behavior closer to that of metallic systems. These characteristics may contribute to faster charge–discharge dynamics and improved rate performance in the Ca-intercalated configuration. The bandgap is reduced from 2.2 eV in LiWS_2_ to 1.84 eV in CaWS_2_, as shown in Figure 2c,d. This narrowing aligns with the DOS analysis and supports the interpretation of the semimetallic behavior of CaWS_2_. Together, the increased state density and reduced bandgap suggest enhanced electronic connectivity across the layers, facilitating interlayer charge transport and promoting greater carrier delocalization. This conductive environment favors both the charge transfer and redox kinetics in CIBs.

The charge-transfer behavior was evaluated quantitatively based on the oxidation-state-derived charge analysis and electron summation, as summarized in Table 2. The Ca^2+^-intercalated WS_2_ cluster showed greater total electron transfer (1.04 eV^−1^·unit cell^−1^) compared to the Li^+^-intercalated case (0.26 eV^−1^·unit cell^−1^). This enhanced charge donation is consistent with the higher formal charge and polarization characteristics of Ca^2+^. As a result, electron density on W atoms increases (1.06 vs. 0.61 eV^−1^·unit cell^−1^), and charge localization on S atoms becomes more pronounced (−0.51 vs. −0.31 eV^−1^·unit cell^−1^). These shifts imply a more significant redistribution of electron density across the host lattice. Additionally, the OCV of CaWS_2_ was calculated to be 0.37 V, while that of LiWS_2_ was 0.21 V. This suggests a deeper insertion potential and stronger thermodynamic driving force for calcium storage under the given model conditions. These OCV values are theoretical voltages calculated from the total energy difference between the pristine and fully intercalated WS_2_ structures. They reflect the equilibrium potential under idealized conditions using desolvated Li^+^ or Ca^2+^ ions in the gas phase and one mole of ion insertion per formula unit. These voltages do not correspond to experimentally measured open-circuit potentials, which are typically recorded prior to ion intercalation and are influenced by electrolyte composition, interfacial phenomena, and reference electrode conditions. Rather, the calculated values provide comparative insight into the relative favorability and thermodynamic driving force of Li^+^ versus Ca^2+^ intercalation into WS_2_.

Altogether, these electronic and electrochemical distinctions between LiWS_2_ and CaWS_2_ indicate that Ca^2+^ intercalation not only alters the band structure characteristics but also enhances the charge compensation mechanisms within the lattice. These findings support the view that CaWS_2_ is a promising material for multivalent-ion battery electrodes.

### 2.3. Electronic Charge Redistribution and Band Structure Evolution in Intercalated WS_2_

**Figure 3:**
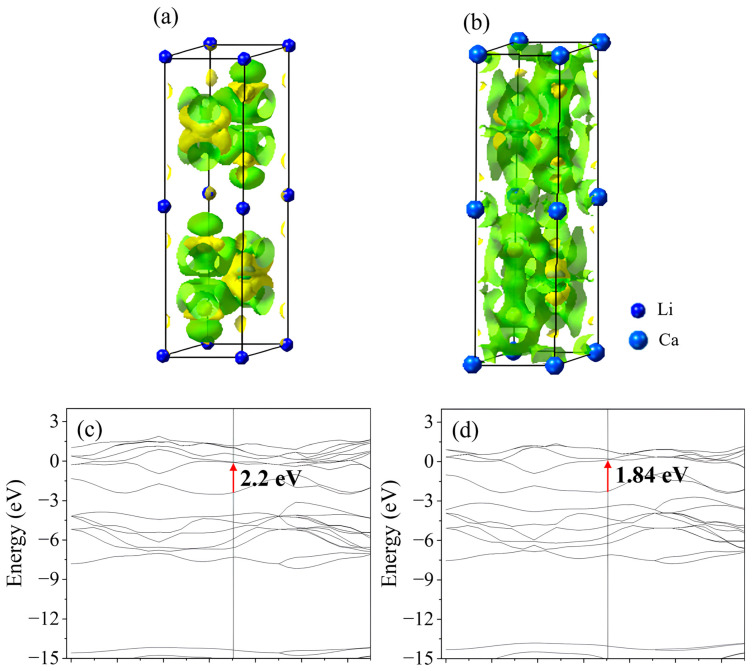
Electronic structures of Li^+^- and Ca^2+^-intercalated WS_2_ configurations. (**a**,**b**) Differential charge-density maps of LiWS_2_ and CaWS_2_, with the green and yellow isosurfaces denoting electron accumulation and depletion, respectively. CaWS_2_ displays a broader charge redistribution across the interlayer region. (**c**,**d**) Band-structure calculations reveal a reduced bandgap in CaWS_2_ (1.84 eV) compared to LiWS_2_ (2.2 eV), suggesting enhanced electronic conductivity. Blue spheres denote the intercalant ions (Li in (**a**) and Ca in (**b**)). In (**c**,**d**), the red arrows indicate the band gap between the valence band maximum and the conduction band minimum.

To better understand the mechanism underlying energy storage enhancement by ion intercalation, electronic charge redistribution and band structure evolution have been investigated [39,40]. The analysis is based on VASP calculations performed on supercell models constructed with a 2H-type layered framework. Differential charge-density maps and electronic band structures of Li- and Ca-intercalated WS_2_ were evaluated to compare their interatomic electron exchange characteristics and charge transport properties. In Figure 3a,b, the isosurface maps illustrate the spatial distributions of electron accumulation (green) and depletion (yellow). Compared with LiWS_2_, CaWS_2_ exhibits more extensive and intensified charge accumulation, particularly around the Ca–S and Ca–W bonding environments. The isosurface contours of CaWS_2_ spread into the adjacent W–S layers, suggesting a broader charge redistribution beyond the local bonding region. This pattern suggests not only localized charge transfer but also spatial redistribution, which may reduce the local strain and improve interlayer cohesion. This redistribution indicates stronger electron donation from Ca^2+^ ions to the lattice, possibly enhancing charge compensation and contributing to structural stability during redox cycling.

Figure 3c,d show the calculated band structures of LiWS_2_ and CaWS_2_, respectively. A clear narrowing of the bandgap is observed upon Ca^2+^ intercalation, with values decreasing from 2.2 eV (LiWS_2_) to 1.84 eV (CaWS_2_). The conduction band of CaWS_2_ also displays a greater curvature near its minimum, implying a reduced effective mass and enhanced carrier mobility. Furthermore, the more gradual evolution of the band edge in CaWS_2_ indicates a reduced sensitivity to structural disorder or dopant-induced perturbations. These electronic characteristics, which include a narrower bandgap, higher dispersion, and enhanced band-edge continuity, may collectively enhance electronic conductivity and improve rate performance. The increased density and dispersion of the conduction band states near the Fermi level are consistent with earlier DOS analysis. These results are consistent with the improved electrochemical behavior of CaWS_2_.

These results indicate that Ca^2+^ intercalation not only modifies the band topology and charge distribution but also introduces electronic features such as dispersive conduction bands and deeper electron penetration. Although LiWS_2_ demonstrates acceptable host characteristics, the enhanced carrier delocalization and improved electronic transport properties observed for CaWS_2_ underscore its potential as a highly promising candidate for CIB applications.

### 2.4. Orbital Hybridization and Lattice Stabilization in Intercalated WS_2_

**Figure 4:**
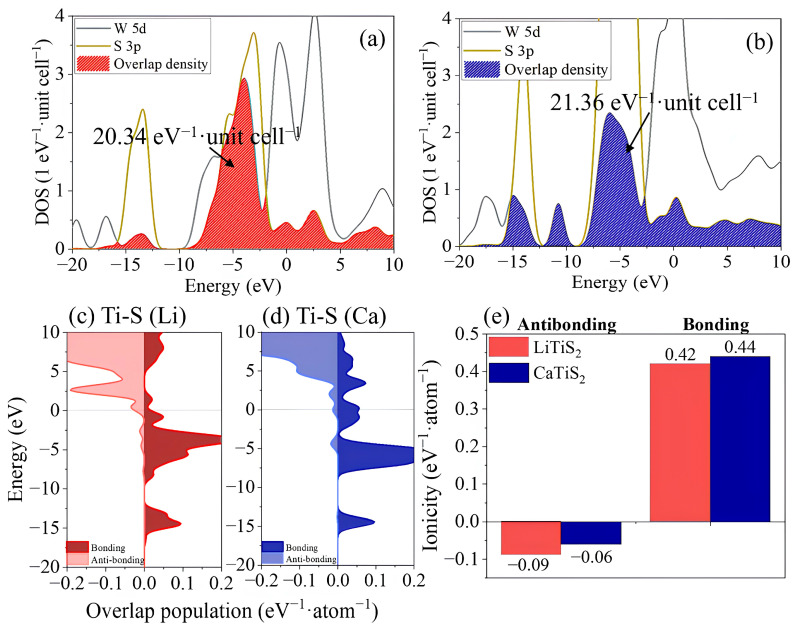
Electronic bonding characteristics of Li^+^- and Ca^2+^-intercalated WS_2_. (**a**,**b**) PDOS and W 5d–S 3p orbital overlap density, with CaWS_2_ exhibiting greater overlap (21.36 vs. 20.34 eV^−1^·unit cell^−1^); (**c**,**d**) energy-resolved overlap populations show a broader bonding distribution and suppressed antibonding states in CaWS_2_; (**e**) integrated values indicate enhanced covalent bonding and reduced destabilizing interactions, supporting improved structural robustness in CaWS_2_.

Understanding the underlying nature of the bonds between host atoms and intercalated ions is critical for elucidating the structural integrity and electrochemical reliability of layered materials. In this section, we explore the effects of Li^+^ and Ca^2+^ intercalation on the electronic bonding characteristics of WS_2_ by focusing on orbital overlap populations (OPs) between W and S atoms. OP analysis provides a direct measure of the orbital hybridization strength. It distinguishes bonding contributions, which stabilize the lattice, from antibonding components, which represent repulsive interactions across the valence and conduction bands. A higher bonding population suggests stronger covalent stabilization, whereas a reduced antibonding contribution corresponds to suppressed repulsive interactions. Both are key for maintaining lattice robustness during prolonged charge–discharge operations [41,42,43]. As illustrated in Figure 4a, the partial DOS (PDOS) and overlap density between the W 5d and S 3p orbitals were plotted for LiWS_2_. The integrated shaded region under the overlap density curve yields a total W–S overlap value of 20.34 eV^−1^·unit cell^−1^, centered near −4.5 eV. This indicates a moderately strong hybridization and suggests that the framework offers adequate structural coherence to accommodate Li-ion diffusion. By contrast, Figure 4b reveals a notably stronger orbital overlap in CaWS_2_, with a total value of 21.36 eV^−1^·unit cell^−1^, a 5.0% increase relative to LiWS_2_. The broader energy span of the orbital overlap in CaWS_2_, particularly between −10 and −2 eV, suggests enhanced multi-level orbital hybridization. This broadened profile may activate deeper and more extended bonding interactions, promote charge delocalization, and improve mechanical coupling between the layers. These combined effects contribute to greater structural resilience under electrochemical stress.

To further assess the bonding environment within WS_2_ following Li^+^ or Ca^2+^ intercalation, the energy-resolved OP between the W 5d and S 3p orbitals was analyzed (Figure 4c,d). The distributions are separated into bonding (right) and antibonding (left) components across the energy axis. This separation illustrates the contribution of orbital interactions to both mechanical cohesion and electronic transport. As shown in Figure 4d, the bonding states for the W–S interactions in CaWS_2_ were not only stronger in magnitude but also spanned a broader energy range than those in LiWS_2_ (Figure 4c). CaWS_2_ bonding orbitals are distributed from −12 to −2 eV, whereas those in LiWS_2_ are more localized around −5 eV. This deeper and more continuous bonding in CaWS_2_ implies stronger orbital participation, which likely improves lattice cohesion and lowers the risk of bond breaking during cycling.

Figure 4e presents a quantitative comparison of the bonding and antibonding electron populations. The bonding population of CaWS_2_ reaches 0.44 eV^−1^·atom^−1^, slightly higher than 0.42 eV^−1^·atom^−1^ in LiWS_2_. Meanwhile, the antibonding population is lower in CaWS_2_ (−0.06 eV^−1^·atom^−1^) than in LiWS_2_ (−0.09 eV^−1^·atom^−1^), indicating a reduced repulsive contribution. Bonding populations > 0 reflect net stabilizing interactions, whereas negative antibonding values indicate destabilization. The decrease in the antibonding population suggests improved structural resilience under ionic stress. In particular, CaWS_2_ shows a suppressed antibonding density in the 0 to +3 eV region, implying fewer repulsive states near the conduction band. In contrast, LiWS_2_ exhibited broader antibonding contributions above the Fermi level, suggesting a higher susceptibility to bond weakening. The simultaneous increase in bonding and decrease in antibonding components in CaWS_2_ reflects a shift toward stronger covalency. This may compensate for the relatively weak electrostatic binding of the divalent ions. Combined with a 33% reduction in the antibonding density, these changes lowered the internal repulsion and enhanced electronic cohesion. This, in turn, may suppress defect formation and amorphization during prolonged cycling.

Taken together, Ca^2+^ not only forms stronger bonding interactions with the W–S framework than Li^+^ but also reinforces the lattice through enhanced orbital hybridization and reduced antibonding character. At the same time, it mitigates destabilizing interactions, leading to an enhanced overall lattice stability. These insights are consistent with earlier charge density and DOS analyses, in which CaWS_2_ exhibited a greater charge redistribution and narrower bandgaps. The current OP-based findings complement these results by connecting Ca-induced bonding rearrangements to improve mechanical properties. Accordingly, Ca^2+^ appears to play a dual role as a mobile guest ion and a stabilizer of the host lattice. Rather than relying on ionic binding, it promoted orbital connectivity and a more even distribution of electronic stress. Although these findings are based on finite-cluster models, they provide meaningful local bonding insights that align with periodic structural trends. This dual behavior may be advantageous for multivalent-ion batteries, where structural robustness under high ionic loads is essential. Overall, the increased orbital overlap, enhanced bonding, and suppressed antibonding in CaWS_2_ suggest a lattice stabilization mechanism uniquely enabled by Ca^2+^ intercalation. These characteristics support the potential of CaWS_2_ as a negative electrode material for CIBs with improved structural durability and extended cycle life. While these insights are derived primarily from DV-Xα analysis, which focuses on localized bonding environments, their consistency with DFT-calculated band structures and experimental cycling data strengthens the credibility of the proposed stabilization mechanism. This synergy between multiscale theoretical approaches reinforces our interpretation of Ca^2+^-induced lattice resilience and provides a solid foundation for examining dynamic ion-transport behaviors in the following sections.

### 2.5. Surface Adsorption and Ion Diffusion Pathways in Intercalated WS_2_

**Figure 5:**
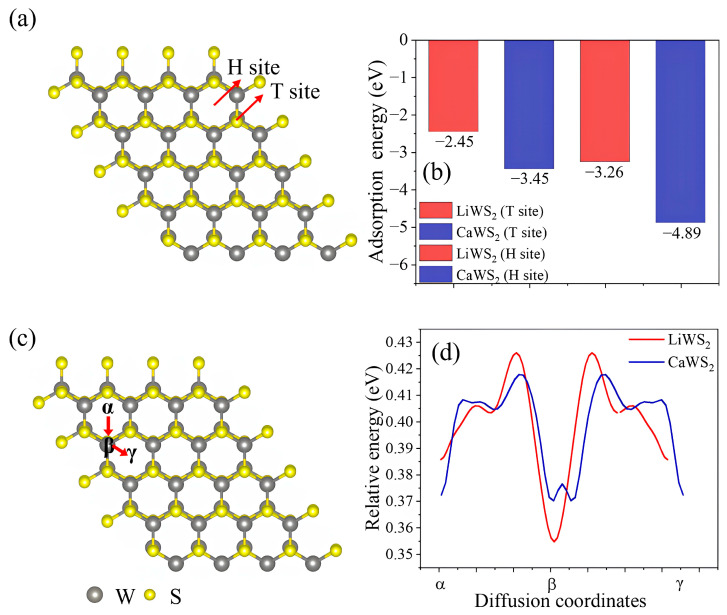
Ion adsorption and diffusion characteristics of Li^+^- and Ca^2+^-intercalated WS_2_. (**a**) Schematic of T and H adsorption sites on the WS_2_ surface; (**b**) calculated adsorption energies show stronger binding of Ca^2+^, especially at the H site (−4.89 eV vs. −3.26 eV for Li^+^); (**c**) three representative ion diffusion pathways (α, β, γ) across the WS_2_ lattice; (**d**) relative energy profiles indicate lower migration barriers for Ca^2+^, implying enhanced ionic mobility despite its larger size. Gray spheres denote W atoms and yellow spheres denote S atoms. In (**a**,**c**), T indicates the top site above a surface S atom and H the hollow site at the center of an S_3_ triangle; α, β, and γ label successive segments of the diffusion coordinate between adjacent adsorption sites (referenced in (**d**)).

Following the evaluation of the electronic bonding characteristics and orbital overlap behavior, the interfacial phenomena governing the ion transport dynamics within WS_2_ were examined. Specifically, the energetics of surface ion adsorption and in-plane diffusion were analyzed because these processes are critical for determining the rate capability and cycling stability of layered negative electrode materials [44,45]. Figure 5a presents a schematic top view of the WS_2_ surface, highlighting two adsorption sites; the T site, located atop a triangle of sulfur atoms, and the H site, located at the center of a hexagonal void. These positions represent energetically distinct environments for guest-ion accommodation. As shown in Figure 5b, the adsorption energies were computed for Li^+^ and Ca^2+^ at both sites. For LiWS_2_, the values are −2.45 eV (T site) and −3.26 eV (H site). For CaWS_2_, stronger adsorption is observed: −3.45 eV (T site) and −4.89 eV (H site). These results indicated that Ca^2+^ exhibited stronger binding at the surface, particularly at the H site. This suggests a more stable interfacial configuration and greater resistance to desorption during the early-stage intercalation. The notably stronger binding of Ca^2+^ at the H site (−4.89 eV vs. −3.26 eV for Li^+^) reflects more favorable electrostatic and polarizable interactions at the surface cavity center. This thermodynamic stability may help retain Ca^2+^ under operating conditions, suppress interfacial degradation, and support long-term cycling. In practical terms, this level of surface binding may enhance interfacial contact and contribute to improved electrode durability.

In addition to static adsorption, the dynamic ion-migration behavior was assessed to evaluate the in-plane transport properties. Figure 5c illustrates three representative diffusion pathways within the WS_2_ lattice: α (T → H), β (T → T), and γ (H → H), encompassing both axial and lateral migration. As shown in Figure 5d, the energy profiles of these pathways revealed clear distinctions between the two systems. For the α path, the diffusion barrier is 0.17 eV for LiWS_2_ and 0.08 eV for CaWS_2_. Similar trends were observed for the β- and γ-paths, indicating that Ca^2+^ can diffuse more easily despite its larger ionic radius. This counterintuitive result suggests that the lower diffusion barrier of Ca^2+^ is governed less by steric hindrance and more by a flatter potential energy landscape than that of Li^+^. This flattened landscape may have resulted from the reduced Coulombic confinement and a more uniform charge-density distribution around the diffusion pathway. Additionally, the weaker electrostatic and covalent interactions between Ca^2+^ and the WS_2_ framework can facilitate ion migration with minimal structural distortion.

These findings are consistent with previous orbital overlap and charge-density analyses, which showed reduced antibonding character and greater electron delocalization in CaWS_2_. These dual characteristics of strong surface anchoring and facile in-plane diffusion suggest that CaWS_2_ simultaneously suppresses ion desorption and supports rapid charge transport. Such a combination is rare but desirable for high-rate CIB electrodes. Notably, the enhanced ionic mobility of CaWS_2_ did not appear to compromise the lattice integrity. The W–S bonding network remained stable, as supported by deep orbital hybridization.

Overall, the coexistence of strong interfacial adhesion and low-barrier ion diffusion may explain the superior electrochemical behavior of CaWS_2_ compared to LiWS_2_. CaWS_2_ combines high binding strength and rapid ion transport; these two properties are often considered mutually exclusive, while maintaining structural stability. This unusual combination supports its potential as a high-rate, mechanically resilient negative electrode material for next-generation CIBs.

### 2.6. Voltage Profiles and Intercalation Mechanisms in Li^+^- and Ca^2+^-Intercalated WS_2_

**Figure 6:**
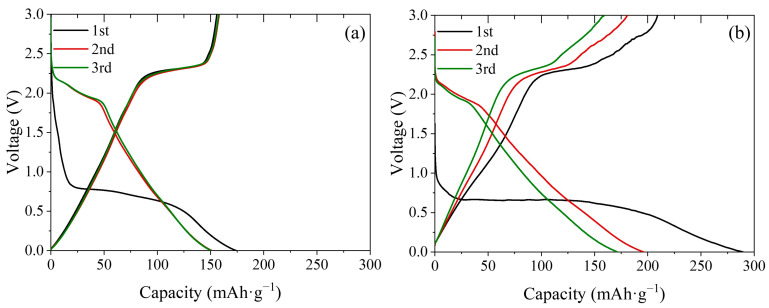
Galvanostatic charge–discharge profiles of WS_2_ electrodes during the first three cycles at a current rate of 0.1C in electrolytes containing either Li^+^ or Ca^2+^ ions. (**a**) Li^+^ insertion (charging) shows a sloped voltage profile with a weak but visible plateau near 0.75 V and a first-cycle discharge capacity of 155 mAh·g^−1^, suggesting a mainly solid-solution mechanism with minor two-phase features; (**b**) Ca^2+^ insertion exhibits a more defined plateau near 0.7 V and a higher first-cycle discharge capacity (208 mAh·g^−1^), consistent with a two-phase-like insertion process.

To experimentally validate the theoretical findings regarding the ionic interactions and charge transport within WS_2_, galvanostatic charge–discharge measurements were conducted using WS_2_ working electrodes in electrolytes containing either Li^+^ or Ca^2+^ ions. As shown in Figure 6, distinct intercalation behaviors are observed for the two systems, particularly in terms of the voltage profile shape and specific capacity. Figure 6a presents the first-cycle charging curve (i.e., ion insertion into WS_2_) of the Li^+^-containing electrolyte. The profile shows a gradual voltage decrease with increasing capacity and a weak but visible plateau near 0.75 V, suggesting a mainly solid-solution intercalation pathway with minor phase-transition features [46,47]. The corresponding first-cycle discharge capacity was approximately 155 mAh·g^−1^. In contrast, Figure 6b shows the charging curve of WS_2_ in a Ca^2+^-containing electrolyte. The curve features a steeper initial slope followed by a well-defined plateau near 0.7 V. This behavior is indicative of a dominant two-phase intercalation mechanism, in contrast to the main solid-solution pathway observed for Li^+^ [20,48]. The discharge capacity of CaWS_2_ in the first cycle reached approximately 208 mAh·g^−1^, which is substantially higher than that of LiWS_2_.

A clear difference was observed between the first and subsequent charge–discharge cycles in both Li^+^ and Ca^2+^ systems. Specifically, the first-cycle voltage curves displayed features that were not present in later cycles: a subtle plateau for LiWS_2_ and a prominent plateau for CaWS_2_. These features significantly diminished in the second and third cycles, suggesting irreversible changes during the initial intercalation. The possible factors include partial lattice rearrangement, interlayer expansion, or the formation of an SEI. Each of these can alter ion transport pathways or reaction kinetics in later cycles. The stabilization of the voltage profiles after the first cycle suggests that intercalation proceeds in a reconfigured state, where structural relaxation and electronic adjustments, such as suppressed antibonding states and enhanced charge delocalization, enable a more consistent electrochemical behavior.

The well-defined plateau observed for CaWS_2_ further supports the interpretation of intercalation-driven phase separation, a phenomenon commonly associated with nucleation and growth mechanisms in layered hosts such as Na_3_V_2_(PO_4_)_3_ and MgMn_2_O_4_ [49,50]. This interpretation aligns with previous studies on multivalent-ion systems, where stepwise voltage profiles were linked to the formation of transient two-phase domains [20,48]. In contrast, the sloped profile of LiWS_2_ and its subtle plateau near 0.75 V are characteristic of a diffusion-controlled solid-solution mechanism. These differences suggest distinct intercalation kinetics and structural responses of the two systems.

**Figure 7:**
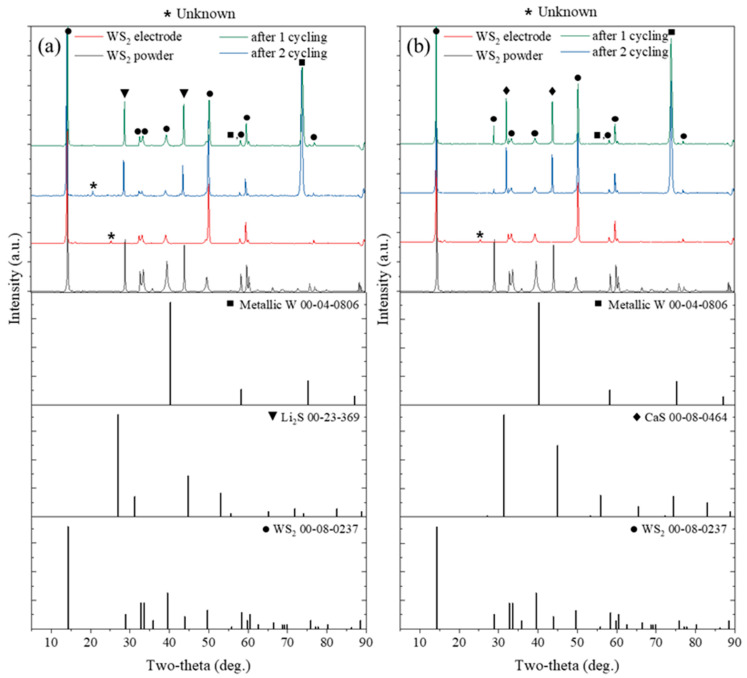
Ex situ XRD patterns of WS_2_ negative electrodes collected at four different states for (**a**) Li-containing and (**b**) Ca^2+^-containing electrolytes (bottom to top: WS_2_ powder, pristine electrode, after first cycle, after second cycle). New peaks that emerge only after cycling are indicated by the following symbols: ▼ Li_2_S; ◆ CaS; ■ metallic W; WS_2_ peaks are denoted by ●; * denotes unidentified peaks. These observations are consistent with partial conversion at deep discharge coexisting with intercalation. Reference patterns: W (00-04-0806), Li_2_S (00-23-0369), CaS (00-08-0464), WS_2_ (00-08-0237).

To examine the structural evolution of the WS_2_ negative electrode during cycling, ex situ XRD patterns were collected before cycling, after the first cycle, and after the second cycle. As shown in Figure 7, peaks that are absent prior to cycling appear only after cycling and are explicitly indicated by the following symbols: ▼ (Li_2_S) in the Li-containing electrolyte, ◆ (CaS) in the Ca^2+^-containing electrolyte, and ■ (metallic W) in both systems, while characteristic WS_2_ peaks (●) remain visible. Because metal sulfides (Li_2_S or CaS) and metallic W are not expected from intercalation alone, these symbol-marked peaks are consistent with the onset of a conversion pathway at deep discharge coexisting with intercalation.

Based on these observations and the prior literature, the electrochemical behavior can be described in two regimes: (i) during the first cycle, the reaction is intercalation-dominant, as supported by the voltage profiles and the retention of major WS_2_ peaks with only incipient conversion signatures [31]; (ii) in subsequent cycles, the emergence of symbol-marked peaks corresponding to metal sulfides and W becomes more evident, whereas WS_2_ peaks persist, indicating that intercalation continues to operate as a principal mechanism [51,52]. Multiple lithiation pathways reported for WS_2_—including intercalation followed by conversion [31,51], conversion-only [53], reversible intercalation-only behavior [54], and coexistence of intercalation and conversion [51,52]—provide the broader context for this interpretation. Overall, our structural trends align with the coexistence scenario and with the theoretical analysis that emphasizes the intercalation regime.

Ca^2+^ intercalation may involve localized lattice distortions and phase-front propagation, whereas Li^+^ insertion proceeds via a gradual continuous interlayer occupation. The stepwise voltage behavior of CaWS_2_ was aligned with the formation of intermediate intercalation phases or lattice rearrangements. This was consistent with the enhanced bonding and reduced antibonding interactions identified in the orbital overlap analysis. In contrast, the smoother voltage response of LiWS_2_ indicates a more uniform and structurally coherent intercalation process. While the plateau in CaWS_2_ supports the phase separation hypothesis, alternative factors such as kinetic trapping, electrolyte decomposition, and solid electrolyte interphase (SEI) formation cannot be ruled out based solely on the galvanostatic data. Additional in situ structural and spectroscopic studies are required to confirm the mechanistic origin of this behavior. Interlayer expansion during Ca^2+^ insertion may further stabilize the host lattice by facilitating structural accommodation despite the larger size of the ion.

Collectively, these voltage characteristics suggest that CaWS_2_ supports a distinct intercalation mechanism with enhanced capacity and electrochemical efficiency. These findings are consistent with theoretical predictions, including a higher DOS near the Fermi level and increased charge delocalization.

### 2.7. Long-Term Cycling Performance and Structural Stability of Intercalated WS_2_

**Figure 8:**
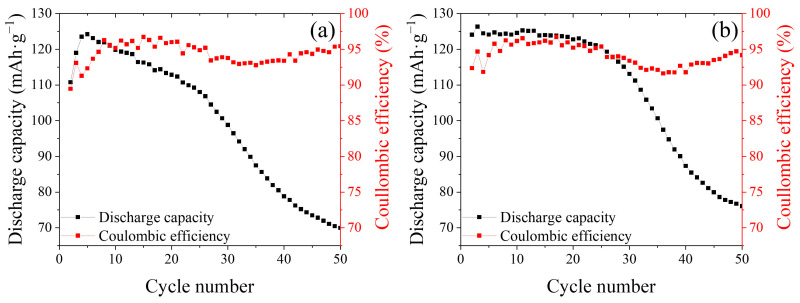
Cycling performance of WS_2_ electrodes at a current rate of 1C over 50 cycles in electrolytes containing Li^+^ or Ca^2+^ ions. (**a**) Li+ intercalation leads to progressive capacity fading, with a retention of 55% after 50 cycles and a Coulombic efficiency above 94%. (**b**) Ca^2+^ intercalation exhibits improved cycling stability, maintaining 61% of its initial capacity and Coulombic efficiency above 95%, suggesting enhanced electrochemical reversibility and structural resilience.

Building on the initial charge–discharge profiles, long-term cycling tests were performed to evaluate the electrochemical durability of the WS_2_ electrodes in electrolytes containing either Li^+^ or Ca^2+^ under extended use. The profiles in Figure 6 were obtained at a low current rate of 0.1C, allowing sufficient time for ion transport and nearly complete utilization of the host structure. In contrast, the data in Figure 8 were collected at a higher rate of 1C, where shortened cycle durations impose kinetic limitations on ion diffusion and interfacial charge transfer. According to Fick’s second law, rapid cycling reduces the diffusion time available for ions to reach deeper active sites, resulting in incomplete intercalation and lower capacity. In addition, high-rate conditions lead to increased overpotentials due to polarization and resistance buildup. These rate-dependent effects are consistent with established electrochemical behavior in layered transition-metal dichalcogenide systems and account for the observed capacity differences. Galvanostatic tests were conducted at a constant rate of 1C over 50 cycles to assess structural and electrochemical stability. The results are shown in Figure 8, which presents the discharge capacity (left y-axis) and Coulombic efficiency (right y-axis) as functions of the cycle number. LiWS_2_ retained approximately 55% of its initial capacity after 50 cycles, indicating moderate long-term stability. The Coulombic efficiency remained above 94%, but capacity fading likely resulted from lithium-site degradation, interfacial reactions, or structural disorder, which is common in layered transition-metal dichalcogenides under extended cycling.

In contrast, CaWS_2_ exhibited 61% capacity retention after 50 cycles, along with a consistent Coulombic efficiency of above 95%. This suggests more stable and reversible calcium intercalation, despite the higher charge density of Ca^2+^. This behavior is consistent with the dominant two-phase-like intercalation mechanism observed during the initial charging, which may facilitate controlled phase evolution and reduce irreversible structural disruption. The comparatively slower capacity fading of CaWS_2_ implies that the host lattice tolerated repeated cycling more effectively. This may be due to the enhanced orbital hybridization and reduced antibonding character at the W–S interface, as shown in the DV-Xα and DFT results. These bonding characteristics likely suppress the structural fatigue and layer distortion by promoting uniform charge redistribution across the interlayer regions. These experimental results are consistent with theoretical predictions. DFT and DV-Xα calculations indicate that CaWS_2_ has a narrower bandgap, a higher DOS near the Fermi level, and stronger W–S bonding, all of which are favorable for enhanced conductivity and mechanical integrity.

Furthermore, the reduced antibonding orbital population supported the hypothesis that CaWS_2_ is less susceptible to lattice disruption under repeated ionic loading. The 6% improvement in capacity retention compared with that of LiWS_2_ further supports the structural resilience of divalent intercalation. This performance advantage may partly arise from the mechanistic difference in ion insertion; Ca^2+^ proceeds via a more distinct phase transformation pathway, whereas Li^+^ intercalation follows a predominantly solid-solution route. Despite the general challenges associated with multivalent-ion insertion, Ca^2+^ can be stored effectively in WS_2_ without severe capacity loss or degradation. This resilience likely resulted from a combination of electronic-level lattice reinforcements and reduced Coulombic repulsion. While these results highlight the superior stability of CaWS_2_, other factors, such as SEI formation, interfacial resistance, and conductivity loss, may also contribute to capacity fading and should be further investigated. Overall, CaWS_2_ has emerged as a compelling candidate for future studies aimed at clarifying the charge–structure interactions in multivalent-ion-intercalation systems.

## 3. Materials and Methods

### 3.1. Computational Methods

#### 3.1.1. Local Electronic Structure and Bonding Analysis via DV-Xα Cluster Modeling

To investigate the ion intercalation behavior in WS_2_ at an atomistic level, finite cluster models were constructed to mimic the local atomic coordination and electronic environment of the layered structure, providing a suitable input for DV-Xα analysis. The cluster consists of 24 W atoms and 48 S atoms arranged to reproduce a representative portion of the WS_2_ framework. Li^+^ or Ca^2+^ guest ions were introduced into the interlayer region to represent intercalation configurations relevant to the actual ion–host interactions, and charge neutrality was maintained by applying formal oxidation-state balancing. The Madelung potential was used to assign partial ionic charges, maintain electrostatic consistency, and capture the ionic nature of the system. All theoretical calculations were performed under the assumption that Li^+^ and Ca^2+^ ions were in a fully desolvated state after insertion into the WS_2_ structure. Solvation effects, including coordination numbers and desolvation energy, were not considered. This idealized model isolates the intrinsic structural and electronic changes induced by intercalation within the host lattice. This cluster-based approach is particularly advantageous for analyzing localized bonding environments, orbital overlap, charge-transfer behavior, and the electronic nature of ion–host interactions using the DV-Xα method.

To elucidate the electronic structure and chemical bonding characteristics of the WS_2_ system during ion intercalation, the DV-Xα method, which is a MO-based, self-consistent field approach within the local density approximation, was adopted. This technique enables high-resolution analysis of orbital-level interactions, allowing for a quantitative and electronically resolved interpretation of ion–host interactions based on detailed electronic structure characteristics, which are critical for understanding electrochemical behavior. In contrast to periodic DFT methods, the cluster-based DV-Xα approach provides localized insights into the bonding environments, charge redistribution, and electronic hybridization effects induced by ion intercalation. Calculations were performed on fully relaxed WS_2_ clusters with intercalated Li^+^ or Ca^2+^ ions, based on previously reported VASP-optimized geometries.

The MO diagrams of pristine and ion-intercalated WS_2_ were analyzed to identify changes in the frontier orbital positions and assess the energy-level shifts associated with intercalation. The bandgap for each cluster was estimated using the Laplace “lvlshm” algorithm, a localized virtual level shift method implemented in the DV-Xα suite, which approximates the electronic excitation threshold and provides insight into the carrier transport properties. To further explore the nature of ion–host bonding, the OP between guest ions (Li^+^ or Ca^2+^) and neighboring W and S atoms was calculated. These overlapping populations were used to distinguish between bonding and antibonding contributions, providing a direct measure of the interaction strength and orbital hybridization. The effective atomic charges and covalent electron populations were also evaluated to quantify the charge-transfer behavior and bonding polarity. These parameters are critical for estimating the activation energy barriers for ion migration and understanding how intercalated ions influence the local electronic environment. As a cluster-based method, DV-Xα does not capture the long-range periodicity or extended band dispersion inherent in crystalline materials. Nevertheless, it provides valuable orbital-level insights into localized bonding environments and charge redistribution, offering a complementary perspective to the periodic DFT for understanding intercalation-induced electronic modifications. Overall, the DV-Xα results yield atomistic insight into the relationship between the electronic structure, bonding character, and ionic mobility in WS_2_, thereby complementing the DFT-based structural data and reinforcing the viability of WS_2_ as a high-performance negative electrode material for multivalent-ion batteries.

#### 3.1.2. Structural Relaxation and Energetic Profiling via First-Principles DFT

First-principles DFT calculations were performed using VASP to perform full structural relaxation of both pristine and ion-intercalated WS_2_ systems. Structural relaxation accounts for the volume changes and lattice distortions induced by ion intercalation and provides a reliable foundation for subsequent analyses. The generalized gradient approximation with the Perdew–Burke–Ernzerhof functional was adopted to treat the exchange–correlation energy, and the projector-augmented wave method was used to describe the electron–ion interactions. A plane-wave energy cutoff of 500 eV was applied to ensure numerical convergence, and a Γ-centered Monkhorst–Pack k-point grid of 9 × 9 × 7 was used for Brillouin zone sampling. Geometry optimization was performed until convergence thresholds were satisfied: 10^−5^ eV for total energy and 0.01 eV·Å^−1^ for atomic forces. The resulting relaxed atomic configurations provided consistent structural inputs for subsequent analyses, including ion-adsorption energies, diffusion barriers, and OCVs.

#### 3.1.3. Adsorption Energetics and Open-Circuit Voltage Estimation

The adsorption energies (*E_ad__s_*) of Li^+^ and Ca^2+^ ions on the WS_2_ surfaces were calculated using the following expression, which has been widely adopted in the surface science literature [55]:

E_*ads*_ = E_*Structure*_ − E_*surface*_ − E_*ion*_, (1)

where *E_structure_* is the total energy of the WS_2_ structure with an adsorbed ion, *E_surface_* is the energy of the pristine WS_2_ surface, and Eion is the energy of an isolated Li or Ca atom in a vacuum. A more negative *E_ads_* indicates stronger thermodynamic stability between the ion and the host surface, contributing to improved interfacial stability. The diffusion energy barrier (*E_diff_*) for each ion was evaluated using the climbing-image nudged elastic band method by computing the minimum energy path between two adjacent adsorption sites [56].

E_*diff*_ = E_*final*_ − E_*initial*_, (2)

where *E_initial_* and *E_final_* are the energies of the initial and final adsorption configurations, respectively. A lower *E_diff_* value corresponds to a more facile ion mobility, which is essential for achieving high-rate capability. Furthermore, the OCV was calculated using a standard thermodynamic relationship [57].

OCV = ((*n* − *m*)E(Li*_n_*WS_2_ or Ca*_n_*WS_2_) − E(WS_2_)*_m_* + (*n*−*m*)E(Li or Ca))/(*n* − *m*), (3)

where E(Li*_n_*WS_2_ or Ca*_n_*WS_2_) is the total energy of the intercalated system; E(WS_2_)*_m_* is the energy of pristine WS_2_; and E(Li or Ca) is the energy of the reference metal atom. Here, m and n represent the number of intercalated ions before and after the insertion step, respectively. This formulation provides quantitative insights into the thermodynamics of ion insertion and associated redox potential. To elucidate the electronic structure and chemical bonding characteristics of the WS_2_ system during ion intercalation, we employed the DV-Xα method, which is a MO-based, self-consistent field approach rooted in the local density approximation. This technique enables the high-resolution analysis of orbital-level interactions, allowing for a quantitative and spatially resolved interpretation of ion–host interactions, which are critical for understanding electrochemical behavior. In contrast to periodic DFT methods, the cluster-based DV-Xα approach provides localized insights into the bonding environments, charge redistribution, and electronic hybridization effects induced by ion insertion. The calculations were performed on fully relaxed WS_2_ clusters with intercalated Li^+^ or Ca^2+^ ions, as derived from prior VASP-based structural optimization. All atomic positions and charge states were carefully assigned based on formal oxidation rules to ensure electrostatic self-consistency throughout the model.

We first analyzed the MO diagrams of pristine and ion-intercalated WS_2_ to identify changes in the frontier orbital positions and assess the energy-level shifts associated with intercalation. The bandgap for each cluster was estimated using the Laplace “lvlshm” algorithm implemented in the DV-Xα suite, which provides a reliable indication of the electronic excitation threshold and its influence on the carrier transport. To further explore the nature of ion–host bonding, we calculated the OP between the guest ions (Li^+^ or Ca^2+^) and neighboring W and S atoms. These overlapping populations were used to distinguish between bonding and antibonding contributions, providing a direct measure of the interaction strength and degree of orbital hybridization.

In addition, we evaluated the effective atomic charges and covalent electron populations to quantify the charge-transfer behavior and bonding polarity. These parameters are critical for estimating the activation energy barriers for ion migration and for understanding the influence of intercalated ions on the local electronic environment. Overall, the DV-Xα results provide atomic-level insights into the interplay between the electronic structure, bonding character, and ionic mobility in WS_2_, complementing the DFT-based structural data and reinforcing the viability of WS_2_ as a high-performance negative electrode material for multivalent-ion batteries.

### 3.2. Experimental Method

#### 3.2.1. Fabrication of WS_2_ Composite Electrodes

Tungsten disulfide powder (WS_2_, ≥99.9%, Sigma-Aldrich, St. Louis, MO, USA), Super P conductive carbon (TIMCAL, Bodio, Switzerland), and poly(vinylidene fluoride) (Mw ≈ 534,000; Alfa Aesar, Haverhill, MA, USA) were used to prepare the working electrode in a weight ratio of 7:2:1. The WS_2_ powder was verified to contain less than 10 ppm of moisture, as determined using a Karl Fischer Moisture Titrator (MKC-710, Kyoto Electronics, Kyoto, Japan). The resulting mixture was dispersed in N-methyl-2-pyrrolidone (Junsei Chemical Co., Ltd., Tokyo, Japan) to form a homogeneous slurry. This slurry was cast onto 18 μm-thick copper foil (UACJ Corporation, Tokyo, Japan) using a doctor blade. The sample was then dried in a vacuum oven (OV-11; Jeio Tech, Daejeon, Republic of Korea) at 80 °C for 12 h. The dried electrodes were punched into 15.95 mm disks and transferred into an argon-filled glove box (SK-G1200, Three-Shine Inc., Daejeon, Republic of Korea) with H_2_O and O_2_ levels below 0.5 ppm. It should be noted that Li^+^ and Ca^2+^ were not pre-doped into the WS_2_ lattice. Rather, ion insertion was achieved via electrochemical intercalation during galvanostatic cycling. The observed structural and electrochemical responses thus reflect the dynamic behavior of WS_2_ upon in situ ion intercalation, not ex situ chemical doping.

#### 3.2.2. Galvanostatic Testing and Electrochemical Evaluation

Electrochemical measurements were performed using CR2032-type coin cells (MTI Corporation, Richmond, CA, USA) assembled in an Ar-filled glove box (SK-G1200, Three-Shine Inc., Daejeon, Republic of Korea). The WS_2_-based composite electrode served as the working electrode, whereas lithium metal foil (Honjo Metal Co., Ltd., Osaka, Japan) was used as both the counter and reference electrodes. Although lithium is not a formal reference electrode in Ca^2+^-containing electrolytes, it has been commonly adopted as a pseudo-reference in multivalent-ion systems due to its relatively stable electrochemical potential. According to Tchitchekova et al. [58], Li pseudo-reference electrodes maintain open-circuit potential stability within ±1–2 mV, while Ca metal pseudo-references exhibit large fluctuations (~40 mV) and potential shifts exceeding 1.4 V. However, it has also been reported that the use of lithium electrodes can lead to Li^+^ contamination of the electrolyte during prolonged testing. In our study, this limitation is acknowledged, and future work will adopt alternative pseudo-reference configurations or three-electrode systems to further isolate Ca^2+^-specific behavior. A microporous polypropylene membrane (Celgard 2400; Celgard LLC, Charlotte, NC, USA) was used as the separator. The electrolytes used in this study were prepared as follows: for Li-ion cells, 0.1 mol dm^−3^ lithium bis(trifluoromethanesulfonyl)imide (LiTFSI, 99%, Sigma-Aldrich, St. Louis, MO, USA), and for Ca-ion cells, 0.1 mol dm^−3^ calcium bis(trifluoromethanesulfonyl)imide (Ca(TFSI)_2_, 99%, TCI, Tokyo, Japan), both dissolved in a 1:1 (*v*/*v*) mixture of ethylene carbonate and dimethyl carbonate (battery grade, ENCHEM, Cheonan, Republic of Korea). All electrolytes were prepared and handled in an argon-filled glovebox, and moisture content was confirmed to be below 10 ppm using a Karl Fischer Moisture Titrator (MKC-710, Kyoto Electronics, Kyoto, Japan). Galvanostatic charge–discharge tests were performed using a battery cycler (WBCS 3000, WonATech, Seoul, Republic of Korea) at room temperature under ambient pressure. Cells were cycled in the voltage range of 0.01–3.0 V at 0.1C and 1C for electrochemical evaluation. Additionally, structural changes in the WS_2_ electrodes before and after cycling were evaluated using X-ray diffraction (XRD). The measurements were performed using a benchtop diffractometer (MiniFlex 600, Rigaku Corp., Tokyo, Japan) equipped with a Cu Kα source (λ = 0.15406 nm, 40 kV, 15 mA). All XRD measurements were conducted at the Advanced Energy and Display Materials Analysis Center of Soonchunhyang University, using equipment registered under the identifier NFEC-2018-12-247471.

## 4. Conclusions

This study comprehensively evaluated the structural, electronic, and electrochemical behavior of WS_2_ under Li^+^ and Ca^2+^ intercalation to assess its suitability as a negative electrode material for CIBs. First-principles VASP and DV-Xα calculations demonstrated that Ca^2+^ intercalation leads to greater interlayer expansion, reduced diffusion barriers, and a narrower bandgap than Li^+^, enabling enhanced ionic mobility and improved electronic conductivity. Orbital-level analyses further revealed stronger W–S hybridization and suppressed antibonding interactions in CaWS_2_, which contributed to increased lattice stability during repeated cycling. Electrochemical measurements validated these computational predictions: the CaWS_2_ electrode achieved an initial discharge capacity of approximately 208 mAh·g^−1^ at 0.1C and retained 61% capacity after 50 cycles at 1C. The voltage profile of CaWS_2_ exhibited a distinct plateau near 0.7 V, in contrast to the sloped curve observed for LiWS_2_, indicating a two-phase-like intercalation mechanism for Ca^2+^ and a solid-solution pathway for Li^+^. This mechanistic distinction likely accounts for the improved structural resilience and cycling performance of CaWS_2_. These results establish WS_2_ as a promising host framework for multivalent-ion storage applications. However, further studies incorporating in situ structural diagnostics, such as operando X-ray diffraction or Raman spectroscopy, are necessary to confirm the intercalation mechanism and identify subtle structural transformations or interfacial changes. Based on the present dataset, the ex situ XRD analysis supports an intercalation-dominant mechanism during the first cycle, which is the regime addressed by the single-phase theoretical model. Under these conditions, conversion-like features at deep discharge are qualitative, and their extent or persistence beyond the first cycle is not established within this study. Overall, this work provides fundamental insights for guiding the rational design of high-performance CIB electrode materials based on layered transition-metal dichalcogenides.

## Figures and Tables

**Table 1:** Calculated crystallographic parameters of pristine WS_2_ and ion-intercalated cluster models. The table lists the structure type, space group, and lattice parameters (*a* and *c*) of W_24_S_48_, (Li_13_W_24_S_48_)^7−^, and (Ca_13_W_24_S_48_)^20−^.

Structure	Space Group	Lattice Parameter (Å)	
		*a*	*c*
W_24_S_48_	P6_3_/mmc (194)	3.15	12.32
(Li_13_W_24_S_48_)^7−^		3.17	13.04
(Ca_13_W_24_S_48_)^20−^		3.53	13.09

**Table 2:** Calculated OCV and electron transfer numbers for Li^+^- and Ca^2+^-intercalated WS_2_ cluster models. Values are provided for guest ions, tungsten, and sulfur atoms based on charge partitioning analysis.

Structure	OCV (V)	Electron Transfer Number (eV^−1^·unit cell^−1^)		
		Guest Ion (Li^+^ or Ca^2+^)	W	S
(Li_13_W_24_S_48_)^7−^	0.21	0.26	0.61	−0.31
(Ca_13_W_24_S_48_)^20−^	0.37	1.04	1.06	−0.51

## Data Availability

Data are contained within the article.
